# Carrageenan From *Kappaphycus alvarezii* (Rhodophyta, Solieriaceae): Metabolism, Structure, Production, and Application

**DOI:** 10.3389/fpls.2022.859635

**Published:** 2022-05-10

**Authors:** Rennielyn Rupert, Kenneth Francis Rodrigues, Vun Yee Thien, Wilson Thau Lym Yong

**Affiliations:** ^1^Biotechnology Research Institute, Universiti Malaysia Sabah, Kota Kinabalu, Malaysia; ^2^Innovation Center, Xiamen University Malaysia, Sunsuria, Malaysia; ^3^Seaweed Research Unit, Faculty of Science and Natural Resources, Universiti Malaysia Sabah, Kota Kinabalu, Malaysia

**Keywords:** extraction method, medical application, physicochemical structure, polysaccharide, production yield, red algae

## Abstract

Carrageenan is a polysaccharide derived from red algae (seaweed) with enormous economic potential in a wide range of industries, including pharmaceuticals, food, cosmetics, printing, and textiles. Carrageenan is primarily produced through aquaculture-based seaweed farming, with *Eucheuma* and *Kappaphycus* species accounting for more than 90% of global output. There are three major types of carrageenan found in red algae: kappa (*κ*)-, iota (*ι*)-, and lambda (*λ*)-carrageenan. *Kappaphycus alvarezii* is the most common kappa-carrageenan source, and it is primarily farmed in Asian countries such as Indonesia, the Philippines, Vietnam, and Malaysia. Carrageenan extracted from *K. alvarezii* has recently received a lot of attention due to its economic potential in a wide range of applications. This review will discuss *K. alvarezii* carrageenan in terms of metabolic and physicochemical structure, extraction methods and factors affecting production yield, as well as current and future applications.

## Introduction

**Figure 1:**
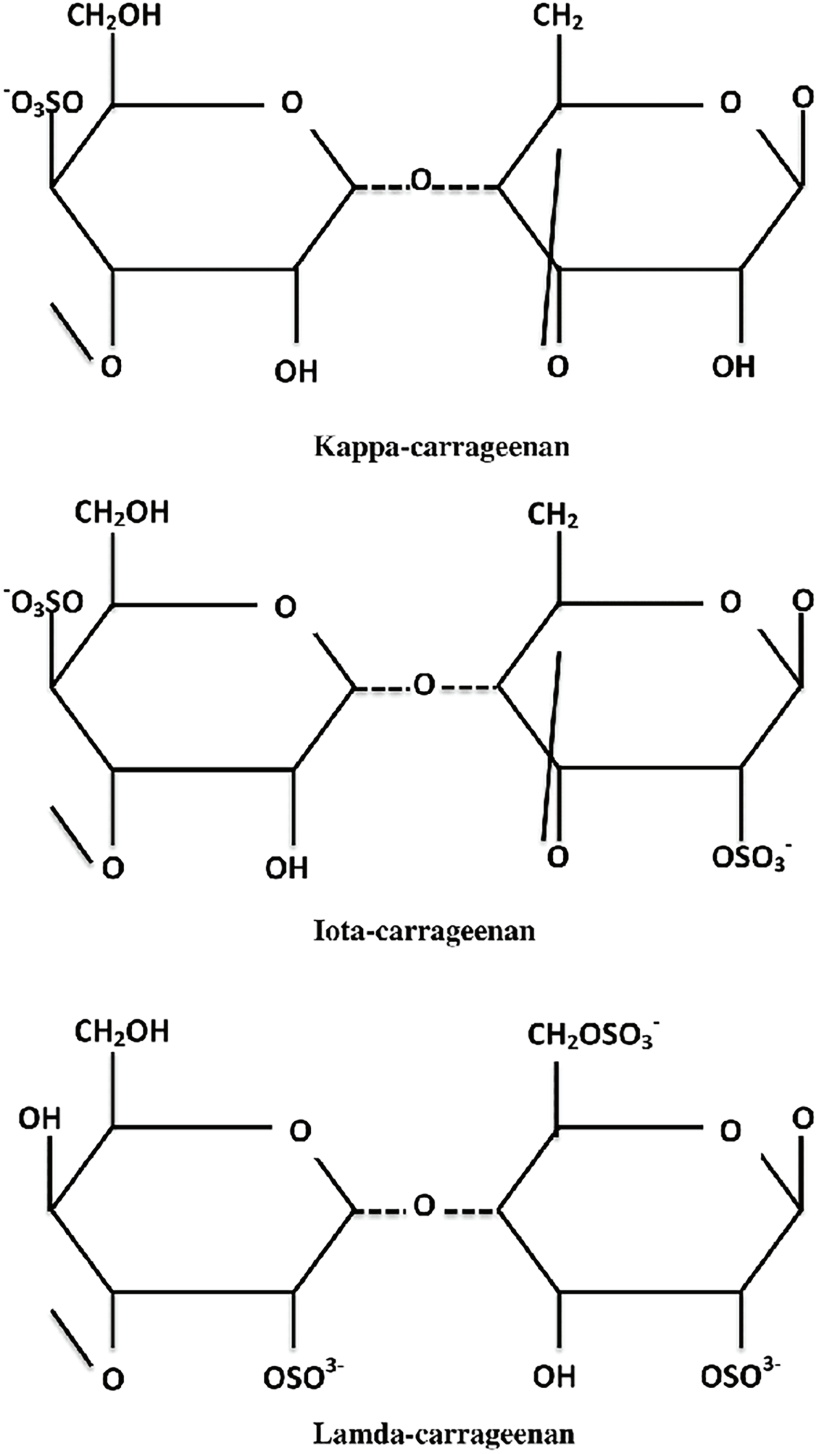
Chemical structures of kappa (*κ*)-, iota (*ι*)-, and lambda (*λ*)-carrageenans.

Carrageenan is a high molecular weight sulfated polysaccharide found in the cell wall of red seaweed, which accounts for a large portion of the marine algal compound market and is used in various industries including food and pharmaceutical (Necas and Bartosikova, 2013; Heriyanto et al., 2018; Guedes et al., 2019; Jönsson et al., 2020). Seaweed cultivation aquaculture produces most of the world’s carrageenan, with *Eucheuma* and *Kappaphycus* species contributing to over 90% of total carrageenan production (Campbell and Hotchkiss, 2017). *Kappaphycus alvarezii*, also previously known as *Eucheuma cottonii*, is a commercially valuable tropical rhodophyte that is highly desired for its cell wall polysaccharide, specifically, the carrageenan, making it the world’s most crucial carrageenophyte (Muñoz et al., 2004; Bindu and Levine, 2011; Lim et al., 2017). On average, 50.8% carbohydrates, 3.3% proteins, 3.3% lipids, 15.6% ash, 12.4% sulfate groups, and 3.0% insoluble aromatics make up *K. alvarezii* (Masarin et al., 2016; Solorzano-Chavez et al., 2019). Carrageenans are classified into three major types (Figure 1): kappa (*κ*)-, which contains only one sulfate group, iota (*ι*)-, which contains two sulfate groups, and lambda (*λ*)-, which contains three sulfate groups (Campo et al., 2009; Subramanian and Varade, 2017). The most important source of carrageenan is thought to be *κ*-carrageenan derived from the red seaweed *K. alvarezii* (Chan et al., 2013; Nurmiah et al., 2017; Syafitri et al., 2017).

The *Kappaphycus* genus becomes one of the essential sources of carrageenan due to its rapid growth rates, with harvest cycles of just 100–120 days, high polysaccharide yields, and a reasonably stable carrageenan composition that is unaffected by the alga’s life history stage (Santhanaraju Vairappan, 2021). The growing demand for carrageenan, which has a wide range of uses, has resulted in a rapid expansion in *K. alvarezii* cultivation worldwide. *Kappaphycus alvarezii* is primarily grown in Asian nations such as Indonesia, the Philippines, Vietnam, and Malaysia and produces relatively pure *κ*-carrageenan. Madagascar, India, Tanzania (Zanzibar), various Central/South Pacific Islands (Kiribati, Fiji, and Solomon Islands), and East Timor are also notable producer countries of this seaweed (Orbita, 2013; Campbell and Hotchkiss, 2017; Kumar et al., 2020). Because of the economic potential of carrageenan, this article addresses several aspects to provide a better understanding of carrageenan metabolism in *K. alvarezii*, updated works of carrageenan production by comparing different latest extraction methods, and applications focusing specifically on the medical properties of carrageenan extracts. This review is intended to contribute to the advancement of the seaweed industry by providing future directions on wider applications of carrageenan to increase its economic potential, in addition to serving as a resource information for academics and researchers working in the field of red algae.

## Carrageenan Metabolism

Polysaccharides are abundant in red algae, including sulfated galactans (carrageenan, agar, and porphyran), structural polysaccharides (cellulose, mannans, and xylans), and storage carbohydrates (floridean starch and *α*-1,4-glucan; Stiger-Pouvreau et al., 2016; Kumar et al., 2020; Sasaki and Yoshikuni, 2022). The total polysaccharide content of red algae ranges from 36 to 76% of dry weight (Holdt and Kraan, 2011). A high percentage of sulfated carrageenans (kappa, iota, and lambda) are found in the cell walls of red algae, and these carrageenans are widely used economically in a variety of sectors (BeMiller, 2019). The *Kappaphycus* species, for example, is crucial since it is the primary source of *κ*-carrageenans, the most demanded biopolymer in the industry (Hotchkiss et al., 2016). While *Kappaphycus* sp. comprises many more components than other seaweeds, the production process is optimized for maximum carrageenan yields. Production of carrageenan in the red seaweed, *K. alvarezii*, is a complicated process, and understanding this process at the molecular level has been a central goal. Hence, this subtopic will focus on the metabolic and biosynthesis pathway and enzymes involved in the carrageenan synthesis in the red algae.

**Figure 2:**
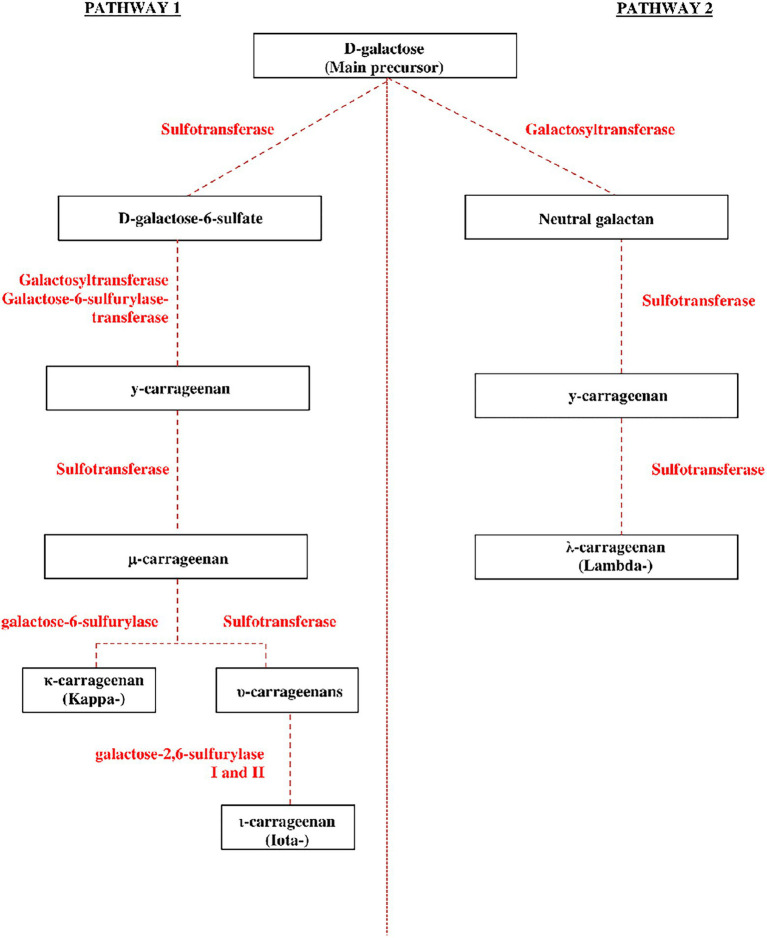
The proposed carrageenan synthesis pathway in red algae (adapted and modified from Cardozo et al., 2007; Ficko-Blean et al., 2015).

It has previously been reported that four main enzymes are involved in the carrageenan metabolic pathway during carrageenan synthesis (Figure 2), namely the sulfotransferases and galactosyltransferases, which are responsible for the early stage of the biosynthesis pathway (Cardozo et al., 2007), and galactose-6-sulfurylase and galactose-2,6-sulfurylase I and II, which are responsible for the later stage of carrageenan biosynthesis (Genicot-Joncour et al., 2009; Collén et al., 2013; Ficko-Blean et al., 2015). The galactose-2,6-sulfurylase I and II enzymes have also been linked to carrageenan biosynthesis, especially catalyzing the conversion of *υ*-carrageenan to *ι*-carrageenan (Genicot-Joncour et al., 2009). Ficko-Blean et al. (2015) established and described two suggested pathways for carrageenan biosynthesis as well as the enzymes involved in the marine red algae. While prior studies hypothesized and developed a biosynthetic mechanism for carrageenan polysaccharide (Wong and Craigie, 1978; Collén et al., 2014), it remains primarily hypothetical owing to a lack of data. According to Ficko-Blean et al. (2015), the carrageenan matrix polysaccharides are assumed to be formed by the cell’s secretory route in the Golgi apparatus, where the enzyme galactosyltransferases form the linear alternating *α*-1,3 and *β*-1,4 linkages of the neutral galactan. Sulfotransferases are thought to catalyze sulfurization in the Golgi apparatus after polymerizing the polysaccharide chain (Muthana et al., 2012). This carrageenan precursor is then suggested to be transported to the cell wall. Here, galactose-6-sulfurylases modify the precursor to generate the 3,6-anhydro-D-galactose molecule (Wong and Craigie, 1978; Genicot-Joncour et al., 2009). To present, this is the only step in the production of algal carrageenan that has been biochemically proven. This pathway was also described in several past publications mentioning the polysaccharide biosynthesis is believed to occur in the Golgi at an early stage in the biosynthesis, while other secondary metabolisms may occur later at the algae’s cell wall. Migration out of the Golgi into the cell-wall matrix occurs at some point during biosynthesis, and additional alteration of the agar polysaccharides can occur as the new tissue matures (Hemmingson et al., 1996; Cardozo et al., 2007).

Carrageenan’s polymer backbone has *β*-1,4 and *α*-1,3 links, indicating coordinated activity of at least two galactosyltransferase enzymes for the second hypothesized pathway. While a neutral galactan has been proposed as the initial precursor for carrageenan (Ciancia et al., 2020), this polysaccharide has yet to be confirmed in red algae. In the precursor galactan chain, the presence of a 6-sulfate group on the *α*-1,3-linked galactose residues is systematic, implying that additional sulfurylations by endo-, random-acting sulfotransferases (prior to the action of galactose6-sulfurylases) are necessary (Wong and Craigie, 1978). While neutral *β*-1,4-linked galactose has been identified in carrageenan (Greer and Yaphe, 1984; Chiovitti et al., 2008), there has been no experimental evidence for neutral α-1,3-linked galactose. Ficko-Blean et al. (2015) proposed two alternative carrageenan biosynthesis pathways. Pathway 1: Instead of starting with a neutral galactan polymer, a 6-sulfated polysaccharide is used to synthesize the precursor galactan chain. As a result, a specific galactosyltransferase joins an activated D-galactose-6-sulfate molecule α-1,3 to the growing mixed-link galactan chain. The alternating β-1,4-linked D-galactose residue is incorporated into a separate galactosyltransferase. Pathway 2: Specialized α-1,3/β-1,4 galactosyltransferases generate a neutral galactan chain, while a highly processive sulfotransferase adds the C6-sulfate group to form D-galactose-6-sulfate, in which this pathway is a refined version of the Wong and Craigie (1978) method.

Following the two metabolic pathways described above, carrageenan biosynthesis can progress to the formation of functional carrageenan. The primary precursor generated after forming D-galactose-6-sulfate molecules is *γ*-carrageenan (Ficko-Blean et al., 2015). Several enzymes catalyze the biosynthetic pathways that convert the precursor γ-carrageenan into more specific and functional carrageenans (kappa-, iota-, and lambda-). The carrageenan conversion process consists of three steps: First, the sulfotransferase converts *γ*-carrageenan to *μ*-carrageenan, and then, the galactose-6-sulfurylase converts the *μ*-carrageenan to the final *κ*-carrageenan (kappa). In the second stage, *γ*-carrageenan is transformed into *μ*- and *υ*-carrageenans by sulfotransferase and galactose-2,6-sulfurylase I and II then convert υ-carrageenan to *ι*-carrageenan (iota). In the final stage, the sulfotransferase converts *γ*-carrageenan directly to *λ*-carrageenan. This assertion is confirmed by a previous study in which *μ*- and υ-carrageenans are the biological precursors of *κ*- and *ι*-carrageenans, respectively, and this conversion is catalyzed by the enzymes sulfotransferases and sulfohydrolases (Antonopoulos et al., 2005; Van de Velde et al., 2005). The previous study indicates that the fundamental metabolic processes of red algae are remarkably similar to those of other algae species (Stiger-Pouvreau et al., 2016).

## Carrageenan Structure and Physicochemical Properties

Carrageenan is a class of hydrophilic polysaccharides composed of alternating 3-linked and 4-linked D-galactose residues modified by 3,6-anhydro bridges and substitution with ester sulfate, methyl, or pyruvate groups (Barros et al., 2013; Dave and Gor, 2018; Goel et al., 2019). A variety of additional carbohydrate residues can be found in carrageenan compositions, such as galactose, sulfate, xylose, glucose, and uronic acids (Van de Velde et al., 2002; Knudsen et al., 2012). The presence of 3,6-anhydrogalactose units and the sulfation pattern of carrageenan are believed to contribute significantly to the variation in their structural composition. Carrageenans from most carrageenophytes are rarely pyruvylated or methoxylated, and they have little to no branching but complex substitution patterns (Ciancia et al., 2020). Some carrageenans may contain not just sulfate groups but also other substitutes. For instance, de Góes and Reis (2012) showed a minor quantity of 6-*O*-methyl-D-galactose in the *κ*-carrageenan obtained from *K. alvarezii*, indicating that this situation is possible. The structural alterations in these carrageenans will probably impact their physical and rheological characteristics (Melo et al., 2002; Freile-Pelegrin and Murano, 2005; Andriamanantoanina et al., 2007). Because of the presence of 3,6-anhydrogalactose in their structure, *κ*-carrageenan derived from *K. alvarezii* has been reported to have the highest gelling capacity of all carrageenans (Wijesekara et al., 2011; Mohamed et al., 2012), resulting in opaque, brittle gels with poor freeze–thaw stability (Schefer et al., 2015). Previous research has also shown that 3,6-anhydrogalactose is required to form a gel network, and its concentration is generally connected to the gelling capacity of the galactan, which influences the gel strength of the polymer (Dickinson et al., 2003; Tuvikene et al., 2007). The high gel strength produced by *K. alvarezii* var. Tambalang Green was also described as being most likely attributable to the high 3,6-anhydrogalactose content or the high ratio of *κ*-carrageenan in the variant (Ilias et al., 2017). However, further research is required to confirm this notion because various other elements can influence the gelling ability of red algae.

Natural polymers are extensively employed in various industrial, environmental, and commercial applications to gel, thicken, emulsify, and stabilize solutions due to their capacity to produce thermoreversible gels (Saha and Bhattacharya, 2010; Rode et al., 2018). The number and position of the ester sulfate groups on the repeating galactose units are the key distinctions determining *κ*-, *ι*-, and *λ*-carrageenans (Kariduraganavar et al., 2014; Chauhan and Saxena, 2016). The sulfate content of native carrageenans varies per disaccharide unit. The differences in sulfate content could also be explained by the fact that different red algae species develop distinct forms of carrageenans throughout their developmental cycle (Lechat et al., 1997). The genus *Gigartina*, for example, generates primarily *κ*-carrageenan during its gametophytic stage and *λ*-carrageenan during its sporophytic stage (Kariduraganavar et al., 2014). The structure of *κ*-carrageenan is composed of 25–30% sulfate ester group and 28–35% 3,6-anhydrogalactose, while *ι*-carrageenan is composed of 28–38% and 25–30%, respectively. The *λ*-carrageenan, on the other hand, lacks 3,6-anhydrogalactose and has 32–39% sulfate ester (Barbeyron et al., 2001). Since *κ*-carrageenan contains the smallest amount of sulfate esters compared to other commercial classes of carrageenan, it is the least negatively charged, allowing for less repulsion for intermolecular hydrogen bonding and, as a result, is more likely to produce a strong film with improved gelling properties (Tye et al., 2018). Another advantage of carrageenans is their capacity to reduce the galactose residue’s hydrophilicity and invert the chair conformation from ^1^C_4_ to ^4^C_1_ due to the presence of 3,6-anhydrogalactose in the polymers, which is particularly beneficial to the gelation characteristics of carrageenans (Rhein-Knudsen et al., 2015). Based on the presence and high 3,6-anhydro-D-galactopyranose content and the small sulfate content, *κ*-carrageenan exhibits an excellent film-forming capacity, consistent with its gelling capabilities (Tye et al., 2018). Overall, the gelling ability of red algae is regarded to be dependent on the presence and amount of 3,6-anhydrogalactose and sulfate content in the red algae.

Another rheological property of carrageenan is viscosity, which is a measure of the rate at which molecules flow due to internal friction. The higher the viscosity, the better the distortion resistance (Mulyani et al., 2021). The viscosity of the generated carrageenan is one of the monitored and regulated parameters used to assess the quality of the seaweed produced, and it must be greater than 5 cP to meet the Food and Agriculture Organization of the United Nations (FAO) quality criteria (FAO, 2014). Mulyani et al. (2021) found a moderate correlation between the physical and chemical parameters at the experimental site and the viscosity of carrageenan extracted from *K. alvarezii* seaweed in the waters of Laikang Bay, Jeneponto Regency, Indonesia. The results show that carrageenan viscosity was much higher when seedlings were planted in moderately shallow waters at 10–20 cm spacings. According to a study conducted by Simatupang et al. (2021) that examined the effects of different culture sites on carrageenan viscosity of *K. alvarezii*, the viscosity of carrageenan is primarily influenced by differences in environmental circumstances, particularly temperature and nutrient levels, which vary depending on the cultivation sites. Aside from that, it has been observed that even minor changes to the carrageenan extraction method can have an effect on the resulting carrageenan viscosity. Manuhara et al. (2016) discovered a significant variation in viscosity values when different concentrations of potassium chloride (varying from 1.5 to 3.5%) were used in the carrageenan extraction process. As previously reported, the viscosity varied greatly (3.02 to 45.91 cp) in response to the extraction temperature (ranging from 40 to 88°C), with the highest viscosity obtained at 60° (Webber et al., 2012). Overall, the ambient conditions in the cultivation farms, and the parameters used during the carrageenan extraction procedure, such as alkali solution concentration and temperature, will have an effect on the rheological characteristics of the carrageenan derived from *K. alvarezii*, including gel strength and viscosity.

## Carrageenan Production Using Various Extraction Methods

**Figure 3:**
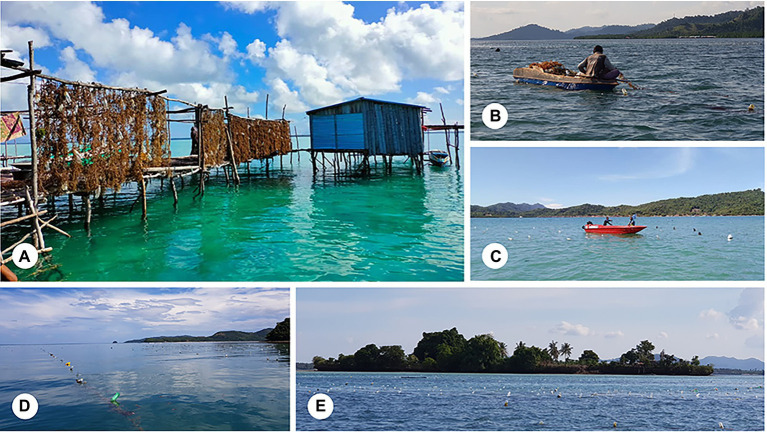
**(A)** A family-operated *Kappaphycus* seaweed farming enterprise; **(B,C)** seaweed farming activities; and seaweed farms on the **(D)** east and **(E)** west coasts of Sabah, Malaysia.

In recent years, there has been a rise in the number of seaweed farms (Figure 3), most notably the cultivation of red algae out of all the numerous types of seaweeds due to their widely used carrageenan compounds nowadays. *Kappaphycus* sp. is a crucial player in the carrageenan industry (Bono et al., 2014), and the manufacturing method is designed to produce the highest possible carrageenan yields (Ortiz-Tena et al., 2017). Depending on the extraction method, carrageenan can be classified into two distinct grades, known as semi-refined carrageenan (SRC) and refined carrageenan (RC; Tarman et al., 2020). Carrageenan is frequently extracted from the seaweed by alkali treatment, and the result is referred to as SRC at this point in the process (Moses et al., 2015). Additional treatments, such as filtering and purifying procedures, are necessary to eliminate residual components such as cellulosic materials, yielding RC (Gunning et al., 1998). The acid-insoluble matter standard differentiates the two varieties of carrageenan that is the essential distinction between them (Tarman et al., 2020). As a result of the various carrageenan extraction techniques presently available, there has been significant debate about which processes produce the most carrageenan yield while needing the least amount of time and money to conduct. This subtopic discusses the available approaches and the benefits and drawbacks of adopting each technique for extracting algal carrageenan from its natural environment.

### Alkaline Treatment Method

When it comes to extracting carrageenan, alkaline treatment is often utilized, and the effectiveness of this procedure is heavily reliant on the type of alkaline solution used. This approach was initially developed for the algae *Chondrus crispus*; however, it is now applicable to all types of algae. Jönsson et al. (2020) recently reviewed this method and published its schematic protocol. In addition to being known as “refined carrageenan,” the original carrageenan is recovered by extracting carrageenan from cleaned and washed seaweed using hot alkaline solutions at temperatures ranging from 95 to 110°C. When a solution such as sodium hydroxide, calcium hydroxide, or potassium hydroxide is added to the seaweed extract for many hours, depending on the kind of seaweed species and size of extraction scales, an alkaline atmosphere can be created (Mishra et al., 2006). Afterward, the residual seaweed matrix that did not dissolve is removed, resulting in a carrageenan solution that is more concentrated than before. The carrageenan may then be precipitated in one of two methods, depending on how much carrageenan is needed. The first method involves the addition of alcohol (mostly isopropanol), while the second involves the formation of gel in a potassium chloride solution (known as the “gel press process”; Lipnizki, 2010). The alcohol-precipitation method can be used to purify all types of carrageenans; however, the gel extraction method can only be used for *κ*-carrageenan (FAO, 2014). Squeezing or freeze-thawing are used to remove the remaining water from the gel after being gelled in the gel press procedure. As a result, the gel press product often has higher potassium chloride concentrations than the alcohol precipitated product. After the carrageenans have precipitated, they must be dried, pulverized, and redissolved to obtain a clear solution of purified carrageenan.

The semi-refining process is a relatively simple type of alkali extraction for carrageenan because it does not retrieve carrageenan from the seaweed matrix. Instead, it treats the seaweed matrix with aqueous potassium hydroxide at around 75°C for 2 h to dissolve and eliminate soluble compounds other than carrageenans, such as salts, soluble sugars, and soluble sugars proteins (Heriyanto et al., 2018). The reagent’s hydroxide component enters the seaweed and reduces the amount of sulfate in the carrageenan while simultaneously increasing the amount of 3,6-anhydrogalactose in the seaweed. The reagent’s potassium component reacts with the carrageenan in the seaweed to form a gel, which prevents the carrageenan from melting in the hot solution. Any soluble protein, carbohydrate, and salts are eliminated when the solution is drained from the seaweed. The residue is rinsed several times to remove the alkali and anything else that might dissolve in the water during the rinsing process. The alkali-treated seaweed is dried for about two days in hot areas before being cut and ground into a powder as SRC or seaweed flour. Bono et al. (2014) investigated the influence of process conditions on the viscosity and gel strength of SRC generated from the red seaweed *K. alvarezii* and determined the optimal conditions for SRC production using alkaline treatment. The optimal alkaline treatment parameters were discovered to be a cooking temperature of 80°C, a cooking time of 30 min, and a potassium hydroxide concentration of 10% w/w, which resulted in a gel viscosity of 1291.84 cP and a gel strength of 94.29 g/cm^2^.

The alkali solution used in both the RC and SRC extraction procedures is intended to improve polysaccharide extraction and accelerate 6-sulfate removal from the monomer form 3,6-anhydro-D-galactose, thereby increasing gel strength and product reactivity on the protein (Corp, 1977). Apart from dissolving the carrageenans, the alkaline solution also transforms the biological precursors *υ*-carrageenan and *μ*-carrageenan into usable *κ*-carrageenan and *ι*-carrageenan, respectively. A cost comparison of SRC and RC technology reveals that the SRC extraction method is less expensive than the RC extraction process because carrageenan precipitation and solvent recovery costs are avoided in the SRC extraction process. However, when compared to the RC, the extracted primary product of the SRC process is of lower quality and is referred to as seaweed flour or alkali modified flour, with the SRC being unsuitable for human food application but designed for pet food production because of the flour is colored and often has a high bacterial population (Rhein-Knudsen et al., 2015). On the other hand, the RC is of higher quality and is referred to as raw carrageenan (Ortiz-Tena et al., 2017). Despite the benefits of alkali treatment, the procedure invariably results in some polysaccharide degradation due to the stresses of the processing environment (heat, alkalinity; Stanley, 1990). It is also possible that a higher concentration of sodium hydroxide will promote carrageenan depolymerization (Varadarajan et al., 2009).

### Enzyme-Based Extraction Method

Several extraction methods have been implemented, with enzyme-based processing techniques being more promising and environmentally friendly than traditional extraction methods (Tarman et al., 2020). Despite the potential for this technology to be used on an industrial scale, there has been relatively little progress in using enzymes to produce carrageenan from seaweed. Regardless, the cellulase enzyme is one of the most widely used enzymes for carrageenan extraction, as demonstrated in *K. alvarezii* (Varadarajan et al., 2009). In general, the enzyme cellulase must be added to the mixture (which includes ground seaweed and distilled water) before boiling it for 1 h in a water bath with a shaker at 50°C. The suspensions were then centrifuged, and the supernatants were separated and used to generate the supernatant fraction. Approximately one volume of supernatant is poured into two volumes of 2-propanol, causing the polysaccharides to precipitate as long fibers. The clear solution was removed from the samples by centrifugation (12,000 rpm, 4°C, 30 min), and the samples were referred to as the precipitated fraction. The samples were dried using a rotary evaporator before being freeze-dried in a freeze dryer. Because carrageenan is found in the hydrosoluble portion of the cell wall component (Lechat et al., 1997), the seaweed’s cell wall must be shattered for the carrageenan to be released. Carrageenans can be effectively extracted using cellulases because cellulose is the primary component of seaweed cell walls (Masarin et al., 2016). Endo-cellulases, exo-cellulases, and *β*-glucosidase are the three major types of cellulose enzymes, and a complete hydrolysis of cellulose microfibrils in the cell wall can be performed by combining these three types of enzymes (Jayasekara and Ratnayake, 2019). Enzymatic hydrolysis can be performed on cellulose in most cases since it is soluble and sensitive to hydrolysis. Furthermore, the absence of lignin in seaweed allows for a faster hydrolysis process (Tarman et al., 2020).

Although this extraction method was considered an alternative to standard extraction (Lechat et al., 1997), the high cost of the enzyme cellulase prevents its widespread use on a commercial manufacturing scale because it is not economically feasible. With a significant decrease in enzyme cost for lignocellulose breakdown, enzyme-based methods may become a viable commercialization option. Besides, the enzyme sulfurylases, which naturally catalyze the transformation of 4-linked *α*-D-galactopyranosyl units into 3,6-anhydro-D-galactopyranoses, have been examined for their ability to convert the precursors into *κ*- or *ι*-carrageenan, which would allow the elimination of alkaline conditions (Genicot-Joncour et al., 2009). However, the viscosity of carrageenan extracted using cellulase treatment was found to be lower than that of carrageenan extracted using standard extraction, which could be due to contaminants present in the extraction process, resulting in a lack of pure carrageenan after alcohol precipitation and centrifugation (Varadarajan et al., 2009). After all, it is still necessary to research ways to improve the method for optimal yield on an industrial scale.

### Microwave-Assisted Extraction Method

Recent efforts to reduce the use of chemicals have resulted in the development of environmentally friendly solvents and approaches that use less solvent and require less energy and extraction time than traditional methods (Ma et al., 2012; Torres et al., 2021). Microwave-assisted extraction (MAE) was used to extract carrageenans from red seaweeds, yielding *κ*-carrageenan from *K. alvarezii* and *ι*-carrageenan from *Eucheuma denticulatum* (Uy et al., 2005). Most seaweed producers prefer sunlight for drying their products because it is less expensive and readily available. However, the technique has a number of drawbacks, including the need for a large amount of land to complete the drying process, being highly dependent on weather conditions, and allowing contamination from the surrounding environment. Microwave drying offers an alternative in SRC extraction because it overcomes the challenges of sun drying and dries biomass materials with a high water content in a shorter time than traditional drying methods (Sari et al., 2020).

The MAE extraction method begins with an alkaline treatment, followed by microwave-assisted extraction using a microwave vessel system at full power and a frequency of 2,450 MHz (Vázquez-Delfín et al., 2014). In brief, 1 g of materials was hydrated for 12 h in 50 ml distilled water (aqueous treatment) and 3% potassium hydroxide (alkaline treatment), and then placed in a closed-vessel system designed to minimize solvent and analyte loss during operation. Extraction can be carried out at high temperatures in closed containers, which accelerates the mass transfer of target carrageenan compounds from *K. alvarezii* samples (Bouanati et al., 2020). Temperature and pressure sensors were used to monitor internal temperature and pressure conditions and to regulate extraction conditions, with a maximum pressure of 159 kPa during the extraction and vessels designed to dissipate excess pressure. Following the completion of the vessel system, the extracted carrageenan was purified and recovered as described above. Nonetheless, Vázquez-Delfín et al. (2014) also reported a decrease in carrageenan yield after alkali treatment in the MAE method, which could be attributed to unavoidable polysaccharide breakdown and loss under rigorous hot alkaline or elevated temperature processing conditions.

### Ultrasound-Assisted Extraction Method

The ultrasound-assisted extraction (UAE) method has been used for decades to extract a wide range of natural raw materials, including from red algae (Cikoš et al., 2018; Bordoloi and Goosen, 2020). This method has recently gained popularity because it requires less equipment and procedures (Tiwari and Troy, 2015), and it is considered an environmentally friendly technology (Talmaciu et al., 2015; Hanjabam et al., 2019). In comparison with other green techniques, ultrasonic technology is significantly easier, takes less time, and is less reliant on the algae biochemical composition as required for enzyme-assisted extraction (Hahn et al., 2012; Zhu et al., 2017b). Youssouf et al. (2017) demonstrated that using the UAE method to extract carrageenans from *K. alvarezii* resulted in higher yields in less time than traditional extraction while not altering the chemical structure of the carrageenan.

The three essential steps of carrageenan extraction using the UAE method are algae pretreatment, carrageenan extraction, and carrageenan purification (Youssouf et al., 2017). The pretreatment process was started by incubating dried seaweed (*K. alvarezii*) in an 80% ethanol solution at room temperature overnight. The pre-treated seaweed was then filtered before being subjected to an ultrasound at 150 W for 15 min to extract the carrageenan. The movement of ultrasonic waves mediates the extraction process, which results in the transformation of ultrasonic waves into mechanical energy. This mechanical energy then ruptures the cell wall, reducing particle size as a result of the rupture and releasing the carrageenan from the cell wall (Nigam et al., 2022). Following the extraction process, the carrageenan was available in the form of a solution, and any remaining algal residues were removed using a hot filtering process. It was necessary to place the filter at 4°C for several hours to obtain carrageenan extract in the form of a gel, and gel carrageenan was frozen and lyophilized to obtain the final carrageenan powder (Youssouf et al., 2017). Ultrasounds allowed for the extraction of 50–55% of carrageenans from red seaweeds such as *K. alvarezii* and *E. denticulatum* in 15 min, and a longer ultrasonic treatment (30 min) may not result in a higher extraction yield (Youssouf et al., 2017; Azis, 2019).

## Factors Affecting Carrageenan Yield and Production

Southeast Asian countries have taken the lead in manufacturing and exporting raw algal material and SRC in the past couple of years, with China and India following closely behind (Hurtado et al., 2014). Red seaweed cultivation increased from 21,000 tonnes in 1950 to 18.3 million tonnes in 2019, accounting for 52.6% of total seaweed cultivation and 47.6% of total value (Cai et al., 2021). In 2019, the world production of carrageenan-containing seaweeds (carrageenophytes) was almost entirely supplied by *Kappaphycus* and *Eucheuma* cultivation in tropical areas, generating a first-sale value of USD 2.4 billion (Cai et al., 2021). Although demand for carrageenan is expected to rise steadily in the coming years, the growth and development of red algae have been hampered by various challenges, including environmental factors that affect carrageenan’s biochemical variability and the emergence of diseases (Setyawidati et al., 2017). It is believed that environmental circumstances (also known as abiotic factors) such as water temperature and salinity, light, water motion, and nutrients impact polysaccharide biochemical contents (proteins, carbohydrates, lipids, fibers, ash, and nitrogen) in polysaccharides (Stengel et al., 2011; Tanniou et al., 2015; Stiger-Pouvreau et al., 2016). The consequences may be immediate, resulting in significant issues with the product quality of the seaweed farming industry. Carrageenan production has also been influenced by biological variables (also known as biotic factors), such as the emergence of the severe disease known as “ice–ice,” the advent of epiphyte outbreaks, and stunted individuals, all of which had an impact on the production yield of *K. alvarezii* (Vairappan, 2006). In a laboratory experiment, Largo et al. (1995) demonstrated that stressful environmental conditions are detrimental to the cultivation of *K. alvarezii*.

### Abiotic Factors

Temperature, light intensity, and nutrition were among the environmental parameters influencing *Kappaphycus* development, with temperature appearing to be the most critical factors determining carrageenan output (Glenn and Doty, 1992). As the temperature rises, so does the yield of carrageenan until it reaches the optimal temperature, at which point the production rate begins to fall rapidly (Bulboa et al., 2008). Temperature variations have also been found to affect the quantity and quality of carrageenan in *K. alvarezii* (Heriyanto et al., 2018). According to Orbita (2013), the maximum carrageenan yield was obtained from *K. alvarezii* when the water temperature was between 28°C and 31°C, but the growth and carrageenan content were very low when the temperature exceeded the optimal range, and the seaweed also died from “ice–ice” disease. Kumar et al. (2020) discovered that seaweeds incubated above 32°C produced significantly lower carrageenan yields than seaweeds incubated at 28°C and that temperatures above the optimal range reduced gel strength and viscosity. Temperatures above 32°C also resulted in lower pigment content and, as a result, lower photosynthetic efficiency, poor growth rates, lower carrageenan yield and quality, and “ice–ice” symptom and biomass loss to thallus fragmentation. Overall, for most *K. alvarezii*, even minor temperature variations (above the ideal temperature) can harm the seaweed’s health, which will undoubtedly impact the production of carrageenan yield.

Salinity is another abiotic factor that may influence the carrageenan yields of *K. alvarezii* because it affects the seaweed’s osmotic equilibrium and nutrient absorption (Aris et al., 2021). Since carrageenan is essential for maintaining the ionic equilibrium of the cell, it is well recognized that salinity impacts the osmoregulation mechanism, affecting carrageenan yield and quality (Percival, 1979). However, Setyawidati et al. (2017) reported that the salinity ranges differing between high (33.8–34.8 psu) and low (33.0–34.0 psu) carrageenan production were only minor, implying that salinity amplitude may be insufficient to explain the variability in carrageenan yield. In contrast, Aris et al. (2021) investigated the effect of different salinity levels (30–34 ppt) on the weight of *K. alvarezii* explants and discovered that the varied salinities affected the growth rate of explants, with the best explant growth occurring at a salinity of 31 ppt and the lowest growth occurring at a salinity of 34 ppt, respectively.

Another factor influencing seaweed carrageenan content is the amount and quality of sunlight that reaches the seaweed. Seaweeds have evolved mechanisms to regulate their photosynthetic activity and adapt to changing light regimes, including the production of pigments that complement the spectral quality of the incident light (Thien et al., 2021). Asni (2021) examined the relationship between seaweed carrageenan yield and season, discovering that the carrageenan content in *K. alvarezii* was higher during the rainy season than during the dry season. However, the study also stated that an increase in sunlight would cause an increase in photosynthetic rates in seaweed and an increase in carrageenan amount in cultured marine algae (Campo et al., 2009). Seaweed cells typically absorb nutrients (carbon, hydrogen, oxygen, sulfate, and nitrate) and convert them into polysaccharides in the form of carrageenan, which can subsequently be deposited on the cell walls *via* the photosynthesis process (Hayashi et al., 2007). As a result, if the photosynthetic activity is disrupted, seaweed development may be hampered, and carrageenan levels may become undesirable (Hurtado et al., 2009).

Some seaweed farms in the southern Philippines, such as Tawi-Tawi, that were once very productive, are now producing only moderate seaweed yields. This phenomenon could be attributed to nutrient depletion caused by poor quality planting material or overstocking of seaweed in farms (Santhanaraju Vairappan, 2021). According to Kumar et al. (2015), nutritional availability is one of the factors that may cause seasonal fluctuations in carrageenan content. Farming practices must be improved to address this issue, such as seedling enrichment with biostimulants to increase growth rate and carrageenan yield (Luhan et al., 2014; Hurtado and Critchley, 2018). The essential macronutrients for seaweed development are carbon, nitrogen, and phosphorus, and it is crucial to managing nutrient availability in seaweed aquaculture or farming to achieve a better outcome (Harrison and Hurd, 2001; Msuya and Porter, 2014). Phosphorus is a significant nutrient that, when combined with nitrogen, plays a vital role in the growth of algae and carrageenan production (Chopin et al., 2004; Sarri et al., 2022). A recent study also discovered substantial seasonal and regional variation in farmed *K. alvarezii* production and product quality in Indonesia, with optimal nutrient availability producing the highest growth and carrageenan yield and quality (Simatupang et al., 2021).

### Biotic Factors

Biotic variables have also been shown to impact carrageenan yield in seaweeds. One of the challenges in seaweed production is regulating epiphytes in farms, which can be detrimental to the overall production. Due to the open nature of the water, other algae have numerous opportunities to travel with the current and adhere themselves as competitors to seaweed that has grown. Epiphytes and diseases are two major environmental factors that can influence the development, output, and carrageenan content of *K. alvarezii* (Syamsuddin et al., 2019). Suboptimal culture conditions, such as low light intensity, low salinity (below 20 ppt), and high temperature (above 35°C), can lead to the development of “ice–ice” disease, which is frequently associated with the growth of epiphytes on the seaweed and can result in lower production or crop failure (Valderrama et al., 2013).

The presence of epiphytes in seaweed production is a common occurrence, but they can become a significant issue that reduces seaweed productivity (Lüning and Pang, 2003; Vairappan, 2006; Hurtado et al., 2007). *Sargassum polycystum* is one of the most common epiphytes attached to seaweed thalli, exacerbating biomass loss in coastal farms (Aslan et al., 2020). Several other epiphytes isolated from the *K. alvarezii* seaweed included *Entheromorpha intestinalis*, *Ceramium* sp., *Neosiphonia apiculata*, *Chaetomorpha crassa*, *Hypnea* sp., and *Gracilaria* sp. (Mulyaningrum et al., 2019). It has been demonstrated that epiphytes cover the surface of seaweed thalli, considerably reducing nutrient absorption (Wakibia et al., 2006; Vairappan et al., 2007). According to Aeni et al. (2019), the combined effects of “ice–ice” disease and epiphyte development decreased the growth rate during *K. alvarezii* cultivation in southeast Sulawesi. Mulyaningrum et al. (2019) compared healthy seaweed to infected seaweed and discovered that healthy seaweed had a higher carrageenan yield and gel strength than infected seaweed. In many cases, the epiphyte was more prevalent and occurred more frequently in conjunction with “ice–ice” disease (Vairappan et al., 2007, 2010; Eranza et al., 2017).

While epiphytes attach to the seaweed thalli, “ice–ice” disease-causing bacteria commonly infect the thalli tips. The bacteria are considered to act as secondary causative agents, but the mechanism by which they do so is currently unknown. Certain studies have shown that the pathogenic bacterium *Vibrio* sp. can quickly adhere to and dominate seaweed tissue as a first stage in the infection process, based on its motile capabilities (Largo et al., 1995, 1998). When exposed to harsh conditions, the bacteria can use carrageenan in seaweed thalli as a carbon source by reaching the medullary layer of diseased branches (Tahiluddin and Terzi, 2021). A similar ability of *Pseudoalteromonas carrageenovora* to produce the specific *κ*-carrageenase enzyme and degrade *κ*-carrageenan in *K. alvarezii* suggested that this bacterium might cause “ice–ice” disease symptoms such as thalli whitening and scaling (Riyaz et al., 2019).

## Carrageenan in Food and Medical Applications

Carrageenan is a food hydrocolloid derived from seaweed that is widely used in the food and cosmetics industries to thicken, stabilize, and gel various products (Noor, 2018; Pereira, 2018). It has been used in the food industry since the 1970s because of its superior gelling and thickening properties (Neish and Suryanarayan, 2017). Carrageenan is now widely used for textural functionality in various products, including dairy, jellies and confectionery, and processed meat products (Hotchkiss et al., 2016). For instance, carrageenan is used in the dairy industry to prevent phase separation, as reported in yoghurt and reconstituted yoghurt (Michon et al., 2005; Ji et al., 2008). Reconstitution at 50°C revealed that 2% carrageenan produced a stable yoghurt product with no visible syneresis for hours, indicating a promising application in the production of long-life yoghurt powder (Pratama et al., 2018). Confectionery jellies containing 1.5% (w/v) carrageenan demonstrated greater hardness, chewiness, cohesiveness, and syneresis, as well as lower melting rates, than gelatin-based jellies (Park et al., 2021). Furthermore, carrageenans aid in the formation of gels and the retention of moisture in meat products, which is especially appealing in low-fat meats because fat reduction frequently results in undesirable, tough textures (Trius et al., 1996). Carrageenans improve the textural properties of meat product by reducing toughness and increasing juiciness, resulting in more desirable sensory characteristics (Nayak and Pathak, 2016).

Carrageenans have grown in importance in recent decades, not only in the food industry, but also in medical, pharmaceutical, and biotechnological research, due to their biocompatibility, high molecular weight, high viscosity, and gelling capacity (De Jesus Raposo et al., 2015). Carrageenans, in particular, have been studied for various bioactive applications, including their role as an antioxidant, antibacterial, antiviral, antitumor, anticoagulant, antihyperlipidemic, and immunomodulatory agent, allowing them to be used as potential pharmaceutical formulations for the treatment of a variety of diseases (Wang et al., 2017; Pacheco-Quito et al., 2020). The following sections discuss some of the important biomedical applications for carrageenans derived from *K. alvarezii*.

### Antimicrobial Properties

The antibacterial properties of seaweeds have opened up a wide range of potential applications in food and pharmaceutical research (Fayaz et al., 2005). *Kappaphycus alvarezii* contains various nutritional compounds, including carrageenan, that can be used as a nutritional supplement. The polymer has shown significant antibacterial activity against a wide range of human pathogens, including *Bacillus subtilis*, *Staphylococcus aureus*, *Lactobacillus acidophilus*, *Escherichia coli*, *Pseudomonas aeruginosa*, *Proteus mirabilis*, and *Vibrio cholerae* (Pushparaj et al., 2014). According to a study, gram-positive bacteria are more vulnerable to seaweed extracts than gram-negative bacteria, and this vulnerability to algal extracts is attributable to the differences in the structure and content of their cell walls (Čagalj et al., 2022). Additionally, *K. alvarezii* carrageenan has shown excellent suppression of *Mycobacterium tuberculosis* (Mayakrishnan et al., 2015). Molecular docking with specific software and visualization tools was used to investigate the mechanism of action of *K. alvarezii* against *M. tuberculosis*. In the study, *κ*-carrageenan was discovered to be able to inhibit the InhA enzyme, which was found to exist in both strains of *M. tuberculosis* and thus controls the activity of the pathogenic bacteria. Besides, oxidized *κ*-carrageenans prepared with hydrogen peroxide were capable of damaging the bacterial cell wall and cytoplasmic membrane, effectively suppressing bacterial growth, and potentially acting as a new antibacterial agent against a variety of food-borne pathogens (Zhu et al., 2017a).When determining antimicrobial activity in polysaccharides derived from seaweeds, criteria such as molecular weight, charge density, sulfated content in the case of sulfated polysaccharides, and structural and conformation features are considered (Pérez et al., 2016). The antimicrobial mechanism induced by macroalgal compounds, including carrageenan oligosaccharides, is generally based on the modification of bacterial cell porousness, which results in the loss of macromolecules, disruption of membrane function, and ultimately the destruction of pathogen cells (Ismail et al., 2020).

### Antiviral Properties

Carrageenan derived from red algae has been shown to possess antiviral properties against a wide range of enveloped and non-enveloped viruses (Ahmadi et al., 2015; Talarico and Damonte, 2016). Since the early 1900s, marine algal sulfated carrageenan has been extensively studied for its antiviral activity, which includes inhibiting viral attachment to the host cell surface, viral internalization and uncoating, and viral transcription and replication (Shi et al., 2017; Álvarez-Viñas et al., 2021). According to Talarico and Damonte (2016), the ability of sulfated polysaccharides to prevent viral entry into the host is the most well-studied biological feature responsible for antiviral action. Due to having the highest sulfate content among the sulfated carrageenans, *λ*-carrageenan has been shown to have better antiviral activity than *κ*- and *ι*-carrageenans (Hans et al., 2021). The chlorosulfonic acid-pyridine approach, sulfuric acid (using a mixture of concentrated sulfuric acid and butanol), and sulfur trioxide-pyridine procedures are commonly used for sulfating polysaccharides to improve their antiviral activities (Ahmad, 2021). The presence of an adequate sulfation degree can be deduced from the fact that sulfation of *κ*- and *ι*-carrageenan increased their activity to levels comparable to those of *λ*-carrageenan; however, no increase was observed after sulfation of *λ*-carrageenan (Álvarez-Viñas et al., 2021). Yamada et al. (2000) expanded on the significance of this feature by showing that desulfation of the original carrageenans reduced their anti-Human Immunodeficiency Virus (HIV) activity. However, previous research discovered that *λ*-carrageenan and *ι*-carrageenan had significantly different anti-HIV potencies despite having comparable sulfate content (Yamada et al., 1997). This finding suggests that antiviral activities are not entirely dependent on sulfate content; instead, the positions and densities of individual sulfate groups on sugar chains appear to be significant considerations (Besednova et al., 2019). Carrageenans are also notable for their ability to inhibit the Human Papillomavirus (HPV) *in vitro*, exerting their inhibitory effects at an early stage of viral infection (Buck et al., 2006). The effectiveness of carrageenan against a variety of sexually transmitted viruses, including the Herpes Simplex Virus (HSV), was discovered to be due to a stiff interaction between the virus and the carrageenan, which causes the viruses to lose their ability to infect cells, effectively reducing virus multiplication and increasing mortality (Carlucci et al., 2002; Bai and Tuvikene, 2021). Shi et al. (2017) described the mechanism of macroalgal polysaccharides against viral diseases during the initial viral attachment phase, where an immune response is triggered when the polysaccharide attaches to virions or the associated protein receptor immunomodulators, activating natural killer cells or inducing an immunological response as part of the process.

### Anticancer Properties

The ability of polysaccharides to successfully inhibit the proliferation and development of cancer cell colonies *in vitro*, and their ability to effectively suppress the growth and spread of malignancies in experimental animals when administered *in vivo*, is still being investigated (Lin et al., 2020). Carrageenans have been shown to suppress tumor cell proliferation and adhesion to diverse substrates by preventing connections between cancer cells and the basement membrane (Pangestuti and Kim, 2014). They also exhibit anticancer activity by improving immunity, which may be related to the activation of the immunocompetence of the body, and specifically targeting key apoptotic molecules, making them potential chemotherapeutic or chemopreventive agents (Yuan et al., 2011; Liu et al., 2019). Calvo et al. (2019) discovered that both *κ*- and *ι*-carrageenans, and carrageenan oligosaccharides such as disaccharides (carrabioses) and disaccharide-alditols (carrabiitols) have cytotoxic effects on LM2 cancer cells, killing them *via* an apoptotic mechanism. Furthermore, previous research has shown that carrageenan can arrest the cell cycle at specific stages, such as the G2 or S phases, indicating a mechanism affecting the tumor cell cycle (Prasedya et al., 2016). These activities have piqued the interest of researchers in recent years because cancer is regarded as one of the most severe diseases, posing a threat to human health and life. Unfortunately, most cancer treatments are currently hazardous, frequently affecting not only tumor cells but also normal healthy cells throughout the body. As a result, contemporary pharmacology and biotechnology are charged with the critical mission of discovering novel, effective, and non-toxic anticancer compounds derived from natural sources. The Joint FAO/WHO Expert Committee on Food Additives (JECFA; 2001) concluded that carrageenans are safe for human consumption and thus a safe alternative to chemotherapy based on numerous toxicological studies conducted on animal models (EFSA Panel on Food Additives and Nutrient Sources Added to Food (ANS) et al., 2018).

## Summary

Carrageenan has established itself as a polysaccharide with numerous applications ranging from food to pharmaceuticals. Carrageenan is primarily derived from the red algae *K. alvarezii*, which is the commercial variety of choice due to its rapid growth and low production cost in tropical coastal waters around the world. Carrageenan is synthesized through a series of complex biochemical pathways primarily catalyzed by sulfotransferases and galactosyltransferases and their derivate enzymes. Carrageenan’s chemical and rheological properties are altered by the addition of sulfate ester groups and anhydrogalactose, which alters its gelling and viscosity properties. Because of its desirable gelling properties, *κ*-carrageenan is particularly useful for industrial purposes. Carrageenan can be extracted using strong alkalies or through enzymatic hydrolysis with cellulases; however, the latter method is not currently commercially viable in an industrial setting. The production of *Kappaphycus* seaweeds is influenced by both biotic and abiotic factors, and the emergence of new pathogens is cause for concern, emphasizing the importance of disease-tolerant strain selection. Global warming and climate change have been shown to have a direct effect on ocean acidity, while eutrophication caused by terrestrial runoff has also been shown to have a direct effect on productivity. Aside from carrageenan, research is becoming increasingly important for identifying clinically important biomolecules as potential substitutes for current pharmaceutical drugs. The emergence of the oceans as the next frontier in agriculture will ensure that carragenophytes remain on the scientific horizon as an economically and environmentally viable option for humanity’s future.

## Author Contributions

WY and KR conceived of the presented idea. RR and VT performed the literature search, data analysis, and drafted the manuscript in consultation with WY and KR. All authors read, provided critical feedback, and approved the final manuscript.

## Funding

This research was funded by the Universiti Malaysia Sabah under the Priority Field Research Grant Scheme (Grant Ref.: SBK0385-2018).

## Conflict of Interest

The authors declare that the research was conducted in the absence of any commercial or financial relationships that could be construed as a potential conflict of interest.

## Publisher’s Note

All claims expressed in this article are solely those of the authors and do not necessarily represent those of their affiliated organizations, or those of the publisher, the editors and the reviewers. Any product that may be evaluated in this article, or claim that may be made by its manufacturer, is not guaranteed or endorsed by the publisher.
